# Implementing a social network intervention designed to enhance and diversify support for people with long-term conditions. A qualitative study

**DOI:** 10.1186/s13012-016-0384-8

**Published:** 2016-02-29

**Authors:** Anne Kennedy, Ivaylo Vassilev, Elizabeth James, Anne Rogers

**Affiliations:** NIHR Collaboration for Leadership in Applied Health Research (CLAHRC) Wessex, Health Sciences, University of Southampton, Highfield Campus, Southampton, SO17 1BJ UK

**Keywords:** Social networks, Long-term conditions, Self-management support, Qualitative

## Abstract

**Background:**

For people with long-term conditions, social networks provide a potentially central means of mobilising, mediating and accessing support for health and well-being. Few interventions address the implementation of improving engagement with and through social networks. This paper describes the development and implementation of a web-based tool which comprises: network mapping, user-centred preference elicitation and need assessment and facilitated engagement with resources. The study aimed to determine whether the intervention was acceptable, implementable and acted to enhance support and to add to theory concerning social networks and engagement with resources and activities.

**Methods:**

A longitudinal design with 15 case studies used ethnographic methods comprising video, non-participant observation of intervention delivery and qualitative interviews (baseline, 6 and 12 months). Participants were people with type 2 diabetes living in a marginalised island community. Facilitators were local health trainers and care navigators. Analysis applied concepts concerning implementation of technology for self-management support to explain how new practices of work were operationalised and how the technology impacted on relationships fit with everyday life and allowed for visual feedback.

**Results:**

Most participants reported identifying and taking up new activities as a result of using the tool. Thematic analysis suggested that workability of the tool was predicated on disruption and reconstruction of networks, challenging/supportive facilitation and change and reflection over time concerning network support. Visualisation of the network enabled people to mobilise support and engage in new activities. The tool aligned synergistically with the facilitators’ role of linking people to local resources.

**Conclusions:**

The social network tool works through a process of initiating positive disruption of established self-management practice through mapping and reflection on personal network membership and support. This opens up possibilities for reconstructing self-management differently from current practice. Key facets of successful implementation were: the visual maps of networks and support options; facilitation characterised by a perceived lack of status difference which assisted engagement and constructive discussion of support and preferences for activities; and background work (a reliable database, tailored preferences, option reduction) for facilitator and user ease of use.

**Electronic supplementary material:**

The online version of this article (doi:10.1186/s13012-016-0384-8) contains supplementary material, which is available to authorized users.

## Background

Policy and practice in relation to long-term conditions management are increasingly directed to a greater focus on the delivery of healthcare in public health spaces. Interventions are being developed that extend to include implementation across a range of settings. Evaluation of the implementation of evidence-based practices pertaining to long-term condition management is increasingly commonplace with more attention being paid to complexities such as multi-morbidity. There has been less focus on evaluation using implementation science methods of co-produced (where design and delivery of services is shared with users) and patient focussed interventions in open and informal healthcare settings [1].

Social interaction and the impact of significant others has been associated with whether or not a self-care regime is followed, and autonomy and control has been identified as having relevance to designing acceptable self-care strategies [2]. Evidence of the positive role social networks have in managing a long-term condition suggests that current self-management initiatives emphasising individual motivation and behaviour change are likely to be enhanced by the development and implementation of strategies for linking people to wider resources through engaging social networks and local support [3–5]. Implementation in patient, domestic and community settings is multi-layered requiring a focus on co-production and the implementation of strategies which cross informal and formal healthcare boundaries. In this paper, we focus on evaluating the acceptability, appropriateness and implementation of a collectively orientated long-term condition self-management strategy. Rather than an exclusive focus on individual self-management, a social network approach aims to enhance the take-up of available and underused collective support. The approach and web-based tool have been designed to improve people’s ability to navigate and negotiate support available from within personal social networks and extend this to engagement with local groups and organisations [6]. This builds on an existing body of knowledge concerning implementation of self-management interventions. Previous research has shown that self-management support is not routinely adopted and implemented in primary care because of the perceived lack of relevance and fit to feasible and recognisable sources of support. Additionally, facilitating sources of self-management support is not viewed as a priority by healthcare professionals [7]. Thus, the tool has been developed to orientate delivery in community and domestic settings where people with long-term conditions are elicited as partners in making health and well-being choices with support from a semi-professionalised workforce.

The practice of self-management for participants is for the most part orientated to the immediacy of present day requirements and the availability of a set of enabling connections, links and activities for managing and living daily life with a condition [7, 8]. Network members located in the personal community of a person with a long-term condition are sources of emotional, practical and illness-related ‘work’ [6]. Where such support is substantive, it can make a major contribution to an individual in maintaining and sustaining a functioning and meaningful life. Conversely, where support is limited, people often report poorer health-related outcomes and a lack of access to resources to help them deal with day-to-day problems, or to understand and manage their condition. Strategies of support which take health literacy into consideration have been called for because of the former’s relationship to improved health status and participation in healthcare [9, 10].

Diverse social networks that extend beyond health professionals and close family to incorporate casual acquaintances, friends and groups seem to provide greater protective health and wellness benefits than those with smaller or family centred networks [11]. A recent review paper suggests three mechanisms are implicated in mobilising network support. Network *navigation* (identifying who should be contacted to make decisions or provide help to access previously unused resources and prioritising access to some ties whilst abandoning others), *negotiation* within networks (reshaping relationships, roles, expectations and terms of engagement and communication between and by network members) and building c*ollective efficacy* (developing shared perceptions and capacity aimed at successful management through shared efforts and objectives) [4, 12].

Access to resources to enable self-management can be built through new connections or reconfiguring the use of existing networks to engage with wider resources embedded in voluntary and community groups and organisations [13, 14]. The intervention discussed here builds on an approach, tested in an RCT, which used tailored information and telephone-guided access to community resources. The RCT demonstrated effectiveness in improving health-related outcomes [15]. Participation in community organisations is consistently related to better health status and improved efforts to self-management [3, 16]. Engagement with such support is likely to be improved by user awareness of existing local groups and reflexion about their suitability. To this end, we developed a web-based tool which takes a multi-level, networked approach to person-centred self-management support which takes these mechanisms into account. The tool (GENIE—Generating Engagement in Network Involvement) maps social networks, allows for user-centred preference and need assessment and facilitates engagement with local support resources.

The aim of this paper is to determine firstly whether the intervention is acceptable and implementable in practice (in a UK setting) and works to enhance support for people with long-term conditions and secondly to add to theory and understanding of how and why knowledge concerning social networks can improve engagement with resources and activities [17].

### The intervention

GENIE has a number of elements which are theoretically and evidence-based (see Table 1 and Additional file 1). GENIE has been developed to be an intervention which is co-produced as the person with the long-term condition has ownership of the network map and links to favoured activities. The facilitator is there to guide them through the process. Facilitators could be from a lay or professional background. The process was designed to take between 30 to 40 min to deliver and consisted of the following stages:

**Table 1:** GENIE elements

Elements	Details	Theory of how it works
Filter questions	The process starts with questions to provide details of the user’s context. This includes postcode; gender; age and health condition.	• Providing filter questions allows tailoring of suggestions and helps to reduce choice at the preference stage.
Concentric circles: Stage 1	Social network members (family, friends, groups, professionals) are represented and mapped, depending on subjective importance, onto three concentric circles. Details of relationship and frequency of contact are recorded.	• To explore everyday relationships and how network members contribute to support • To note change over time • To provide a visual image to enable engagement • To help people become conscious and reflexive of contributions made by others to self-management support (SMS) • As starting point for a discussion about how to extend existing support, access support from new sources, or change existing practice.
	• Support work can be: illness-related (taking medications and measurements, understanding symptoms, making appointments); everyday (housekeeping, child rearing, support for diet and exercise, shopping, personal care); or emotional (comforting when worried or anxious, well-being, companionship).	
Typologies: Stage 1	Feedback and a summary is provided on network types:	• To help people become conscious and reflexive of network structure and availability of SMS • Act as a prompt for healthcare professionals and others to take action where there are obviously fragile networks
	Diverse - family, friends, and community groups with *regular frequent contact*;	
	Friend and/or family centred – mainly friends and/or family members with *regular contact and support*;	
	Friend and/or family contact - some mostly friends and/or family members with *limited or patchy support*;	
	Isolated or professional contacts only	
Preferences: Stages 2,3,4	The user co-produces and owns the network map.	• Non-intrusive methods are more effective than highly directive approaches which often fail because they do not deal with existing relationships to negotiate time and space for new activities (intimidating to attempt by oneself) or needing help with transport • The user is made a capable and willing to reciprocate participant • To reduce choice and complexities arising from information overload counterproductive for learning, social engagement and social support particularly where there is poor health literacy.
	Choices are tailored using a series of questions and based on preference and enjoyment rather than on health-based need. For example, the facilitator prompts by asking:	
	“Are there things you used to do that you don’t do anymore? What stopped you from continuing to do these things?”	
	This gives clues about how to identify the most relevant type of support, the likely barriers they may encounter, and how to encourage them to restart these activities.	
	Network members are selected as potential buddies to accompany them to new activities.	
	Asked to select the three activities or resources they are most interested in and agree to try them out. The locations of the activities are displayed on a Google-based map.	
Links to Voluntary and Community Organisations (VCOs): Stages 2,3,4	The preference questions link to community resources in a pre-created database.	• Diverse networks which include VCOs enhance health and well-being through providing access to new acquaintances for advice, support and links to resources are often missing where there is reliance on strong family ties. • Support from VCOs is non-clinical. • Specific benefits for people who are isolated.
	Categories in the database include: activities and hobbies, health, learning, support, independent living and volunteering	

Filter questions: To tailor results to the individual.

Stage 1: Mapping of the individual’s current social support network using a concentric circles method.

Stage 2: Eliciting values and preferences for activities and support resources.

Stage 3: Linking individuals to prioritised and valued activities and resources (links are to a pre-created database where local organisations and resources have been categorised—see Table 2).

Stage 4: Present options in a user-friendly way with clear details about access.

**Table 2:** Findings at T3 (12 months after GENIE intervention)

User ID^1^		01	02	04	05	06	07	10	11	12	13	14	15
Types of engagement													
Activities	Singing			*									
	Playing guitar					*							
	Writing					*							
	Coffee group			*									
	Quiz team						*						
	Social Club								*				
	Church											*	
Health	Walking			*	*	*		*	*			*	*
	Line-dancing							*					
	Zumba	*											
	Aerobics	*											
	Swimming							*					
	Table tennis						*						
	Pilates						*						
	Wii tennis									*			
	Gym	*									*		
	Health eating			*	*				*			*	
Learning	Family History Society			*									
	Webinars			*									
Support	Befriending service		*						*				
	Diabetes Support Group	*											
	Resource Centre		*										
	Sugar Buddies	*											
	Facebook group	*											
Independent Living	Mobility scooter					*							
	Pendant alarm		*										
Volunteering	Peer support training	*											
	Charity shop work							*					
Other	Phablet										*		
	Walking stick					*							
	Diabetes recipe cards									*			
	Measuring spoons									*			
	Fitbit	*											
	Shopping trolley		*										

^1^ Three participants did not continue beyond T1, ID3 had type 1 diabetes and IDs8 and 9 had changed circumstances

* indicates that a resource or activity was taken up by the individual

The concentric circles technique is initially used to gain insight into the user’s current situation regarding self-management support and who they view as important in the management of their condition [18, 19] and then to further map the people and groups who could potentially provide extended support. An overview of the completed network is provided to check with the user that the network represents their situation either for personal use or to provide a summary to share with health professionals. This included the number of network members and a description of the type of network: diverse, family-focussed, friend-focussed or isolated [20].

In order to simplify navigation and links to activities and support, the preference stage of the tool was designed to closely align to the articulation of the user’s values and interests. Suggestions for health-related activities included exercise or weight-loss groups and things like hobby groups, support for independent living, volunteering opportunities and educational courses.

GENIE has been designed to fit and integrate with everyday life, to be easy to use and to engage people visually providing immediate feedback on their social network via a co-created visible map and a Google-generated map of the local activities and groups they have indicated an interest in.

## Methods

Table 3 outlines the strategy used to implement GENIE. Theories concerning implementation of technology for self-management support have been used to assist the explanation of how new or modified practices associated with GENIE are operationalised by users and how the technology impacts on relationships, fits with everyday life and allows visual feedback [21, 22].

**Table 3:** Implementation strategy

Actors	Partner organisations: My Life a Full Life and Age UK Isle of Wight (see text)
	Facilitators: health trainers and care navigators
	Users: People with type 2 diabetes living in the community
Actions	Facilitators use the social network tool GENIE with their clients to map out social support networks and link people to local resources
Action targets	The organisations: support facilitators; facilitate training process; find key local stakeholders for working group to develop sustainability strategy.
	Facilitators: attend training; use GENIE tool with clients and recruit 15 case study subjects (*n* ≤ 2 per facilitator); share and discuss learning with researchers
	Users: provided with personal GENIE web account; work through GENIE with facilitator; prioritise up to three new activities or resources to try.
Temporality	Assumption that the user will access the chosen activities or resources soon after the delivery of the intervention.
Dose	Facilitated use of GENIE happens once. This includes:
	1. Creating a social network map using a web-based concentric circle tool. 2. Discussion around the type of support shown in the map and activities they used to enjoy but have dropped. 3. Questions concerning preferences. 4. Prioritisation of up to three activities or forms of support. 5. Generation of a map of the chosen activities in relation to the user’s address. The user can access the website through their password-protected personal account at any time to redraw their network map and find new activities or resources.
Implementation outcome affected	1. Users have a more diverse support network. 2. Adoption of the tool by the facilitators as part of their routine assessment of clients. 3. Sustained maintenance and updating of the database of local organisations with input from facilitators and local people. 4. Partner organisations and commissioners can extract data to see where there are gaps in resource provision.

A longitudinal case study design was used to identify the processes and dynamics of delivering the intervention and to capture individual outcomes of the use of the tool over time. Methods included the use of video (to record the baseline facilitated delivery of the intervention), non-participant observation and semi-structured qualitative interviews. The longitudinal design captured changes in social network support over time as establishing new activities may require or result in different support from different people. Following individual case trajectories over a 12-month period with interviews at baseline (T1), 6 months (T2) and 12 months (T3) generated sufficient data to compare, contrast and examine cohort commonalties and differences (see Table 4). Network maps were revisited at T2 and T3 to add or delete network members and to allow participants to move people or groups between circles to reflect changes in their perceived importance.

**Table 4:** Methods

Methods	When	Purpose
Video	• To capture the delivery of the intervention • Post-intervention to elicit reflection and retrospective sense-making	To show participants to help them recall and talk through what they were thinking at certain points during delivery of the intervention
		To capture non-verbal interactions allowing rigorous post-hoc collaborative review on engagement, elements of work and division of labour undertaken by the facilitator and participant
Non-participant observation	• Researcher observed the facilitated intervention and took notes using a framework with emphasis on demonstrating sense-making and buy-in	To identify points where there were difficulties in understanding and engagement with the intervention. Timings were noted so that point in the video could be revisited during video-elicitation
Interviews	• Immediately following intervention (T1) • 2 weeks—phone call (to check on uptake of chosen activities) • 6 months face-to-face plus noting of changes in circles diagram (T2) • 12 months—phone call plus noting of changes in circles diagram (T3)	To answer questions about the intervention’s relevance, acceptability, ease of use, promotion of new insights and potential beneficiaries.
		To allow a longitudinal dimension to capture change

We focussed on identifying a deprived and marginalised setting as an area where people were most likely to benefit from network support strategies [3]. Deprivation is associated with: isolation; poor health literacy; poor access to health resources, information and sources of influences; insufficient social capital; low confidence and higher differentials in power with professionals [23–25]. The Isle of Wight (IoW) was identified as a site for implementation because of unequally distributed resources and limited access to further support. Connections were made with those organising the *My Life a Full Life (MLAFL)* programme (see Table 3) which seeks to deliver integrated care and support on the island for older people with long-term conditions and those with mental health needs to increase independence and inclusion in communities. Thus, the intervention aligned with MLAFL’s programme of delivery and community approach (http://www.mylifeafulllife.com/Vanguard/Vanguard.htm). Meetings were held to decide on ways to deliver GENIE and identify appropriate local organisations (the charity age UK) and potential facilitators (health trainers and care navigators). The latter are relatively new additions to the healthcare workforce [26] and are charged with providing ‘information and support to people about healthy living and how to make healthier lifestyle choices’ and ensuring ‘individuals are engaged and connected with their local community and other organisations to make best use of resources.’

Two training sessions were held for the ‘facilitators’, local managers and commissioners (see Additional file 2 Outline of Training) who were given access and training in how to use the tools needed to deliver GENIE.

The intervention and approach were designed to be generic and thus of use to people with long-term physical and mental health problems as well as those who are isolated and lonely. Here, Type 2 diabetes was selected as an exemplar condition because of its prevalence and impact of self-management requirements on daily life including the nature of relationships (support from a range of sources has been shown to generate collective efficacy for people with diabetes) [12, 27] and likely impact on reducing risks associated with diabetes (for example in relation to food and diet) [28].

### Sample and delivery

The UK sample was one case study in a wider European project involving five other countries with similar samples (giving a total of 90 participants). The UK implementation has been used in this paper as the fullest exemplar of all the elements of GENIE. Case study participants were selected to represent a range of ages and gender. Inclusion criteria were adults over 18 years living in the community with type 2 diabetes; exclusion criteria were those receiving palliative care and those who were unable to communicate in English. Participants were recruited from existing clients of facilitators (*n* = 3), from course attendees on the local diabetes education programme (*n* = 6), through a local diabetes support group (*n* = 4) and through researcher contact (*n* = 2). Delivery of the intervention was at the convenience of the participant and took place in individual homes, the public library, places of work and medical centres.

### Data collection

Data was collected from 15 videos and observations of the facilitator-participant interaction at the point of intervention delivery and from in-depth interviews with participants at baseline, 6- and 12-month post-intervention. Observational notes (by EJ) recorded time points of sense-making and engagement with the intervention [21] and non-verbal interactions between the facilitator and the participant so these could be revisited for video-elicitation purposes in the baseline interview and during the analysis. Observational notes were used as a means of engaging participants to think aloud about how they made sense of or engaged with the intervention at certain points.

The baseline T1 interview explored the participant’s experience of being guided through the intervention in terms of acceptability and accessibility. The researcher used a combination of a semi-structured interview schedule and individualised questions relating to specific observations made during the intervention process. The interview was guided by the following key questions:

- Was the intervention of relevance?
- How acceptable was the mode of delivery and how easy was it to use?
- What new insights were gained?
- How much use is this type of information?
- Who might benefit most from this type of intervention?

Two weeks following the delivery of the intervention, participants received a follow-up phone call from the researcher to obtain initial feedback. Changes to participants’ personal support network and engagement with community activities/groups were ascertained and recorded at T2 by means of face-to-face interviews and again at T3 by means of telephone interviews. All interviews were audio-recorded, fully transcribed and anonymised.

### Analysis

Interview transcripts were coded and categorised the participant’s experience of being facilitated through GENIE and to gain a better understanding of the mechanisms of engagement with the process (T1) as a precursor to thematic analysis.

Video recordings of the intervention delivery were viewed independently by three members of the research team (EJ, IV, AK), coded and discussed in light of the observational notes highlighting engagement and sense-making taken at the time of the intervention delivery. Normalisation process theory was used as a set of sensitising concepts for video analysis [21]. A worked example of how this was used can be found in Additional file 3. This ensured a structured examination of the intervention process and the interaction between the facilitator and the participant, both verbal and non-verbal. Early findings were discussed with facilitators at a meeting to triangulate views and experiences of delivering the intervention. Care navigators produced an independent report on their use of GENIE for the MLAFL programme which was shared with the research team. The research team met to discuss emergent themes, share new insights and talk through case studies in order to ensure a reliable and consistent consensus about whether and how the intervention was working to improve social network support for condition management. Further data from follow-up interviews (2 weeks, T2 and T3) were coded to examine change in networks, behaviour and engagement with activities over time. As corresponding network circle diagrams were created at each successive interview, movement and changes in the networks were tracked, compared and contrasted.

The concepts of relationships, fit and visibility derived from a review [22] resonated with the main themes drawn from the interview and observational data in the GENIE study. These concepts were taken into account in order to focus on the relationships an individual had with the social network members who provided support.

### Ethical considerations

Informed written consent was obtained from participants and facilitators in advance of data collection. The study was approved by the Faculty of Health Sciences Ethics Committee, University of Southampton, Reference number 9380.

## Results

Fifteen case study participants were recruited representing a range of ages (43 to 73) and domestic situations. The majority were male, retired, had below average income and most had a college or university education (see Table 5).

**Table 5:** Demographic overview

ID	Gender	Age	Domestic situation	Employment status	Income (average = £25 K)	Highest educational level
01	Female	51	Divorced, lives with adult son	Full-time work	About average	College
02	Male	70	Never married, lives alone	Retired	Lower than average	School (up to 16 years old)
03	Female	57	Lives with partner	Part-time work, part-time voluntary	About average	University
04	Male	54	Never married, lives alone	Unemployed, actively seeking work	Lower than average	College
05	Male	70	Divorced, lives alone	Retired, part-time voluntary	About average	College
06	Male	68	Married	Retired	Lower than average	University
07	Female	59	Lives with partner	Retired	Lower than average	College
08	Female	66	Never married, lives alone	Retired	Lower than average	College
09	Male	43	Lives with partner	Full-time work	Lower than average	School (up to 16 years old)
10	Female	66	Married	Retired, part-time voluntary	Lower than average	School (up to 16 years old)
11	Male	75	Widowed, lives alone,	Retired	Lower than average	College
12	Female	59	Never married, lives alone	Full-time work	Lower than average	School (up to 16 years old)
13	Male	76	Married	Retired, part-time voluntary	Lower than average	College
14	Male	73	Married	Retired, part-time voluntary	Lower than average	College
15	Male	67	Married	Retired	Lower than average	University

### Acceptability and implementation

GENIE achieved the primary aim of getting participants to take up new activities and illuminated the presence of additional forms of cognitive engagement. In terms of understanding and engagement, both participants and facilitators understood what they had to do. Network maps were created, showed change over time and activities and new resources identified. This method enabled the following of how network members were involved and became engaged in the process, providing new insights and understanding of the work of navigating and negotiating relationships.

### New activities

Nearly all participants in the case study increased engagement with an activity, resource or service during the 12-month period following the intervention. Most activities were new, but some represented the rekindling and uptake of former interests. The most significant activities were some form of walking and healthy eating advice (Table 2). GENIE was not relevant in one instance (a participant who had a very active lifestyle, a diverse network and believed that the onset of her diabetes was not related to lifestyle choices). In terms of relevance and acceptability, participants and facilitators engaged readily with the practicalities and potential of the intervention. Analysis has helped to provide a greater theoretical understanding of what activities were taken up and why.

Whilst technological factors (internet connectivity and competence with using a computer interface) had some initial impact on acceptability, it was other aspects of the intervention that had more lasting effects. The website itself was rarely accessed following the initial intervention—it had served a purpose and was seemingly discarded appropriately. Alongside the concepts of relationships, fit and visibility; themes that emerged in analysis related to positive disruption and reconstruction; temporal change and reflection; and non-threatening but gently challenging facilitation.

### ‘A shock that that’s me’: disruption and reconstruction

The concentric circle diagram had a powerful impact on how a person saw and understood their support network. Participants reacted positively to the network mapping exercise and were quick to engage with and understand the implications of a picture they had helped create showing their central position, surrounded by members of their social network. Most participants had not previously visualised themselves in this way. Some were surprised by the size of their network or by their position in relation to other network members. This sudden, visual feedback could be described as a ‘light bulb’ moment.

*“What it [circle diagram] identified were things that I hadn’t really thought about……I’ve never asked myself the question who am I? Where do I fit in the big circle?.....I was just surprised. I thought is that really me? Is that really what I do? Is that where it all fits? Does it fit? …..I just never joined it all up, never thought about joining it all up. ….. Yes it was a bit of a shock overall that that’s me, because I’ve never looked at me. I don’t know if it’s the right expression to say I’m not interested in me…..I tend to almost be an observer….. I almost look at that circle with me on the very outside”* (ID13)

*P: Well I suppose I was surprised because I’ve never written them down like that, you don’t do you? Your friends and your family, they’re there….. but it’s just a bit of an eye opener when you do write them down and then you think it’s a bit short really.*

*I: Is that what you thought?*

*P: Look like Billy no mates according to that! (ID12)*

Combining this visual representation with a conversation with the facilitator about what the network members did to help them manage their condition led to a re-alignment in thinking about relationships. People were able to explore the underlying dynamics within close family ties and what this represented in terms of importance to them through distinguishing between network members who either promoted or inhibited self-management support. The interactivity with the web tool allowed movement of network members around the circles and from the outset this encouraged thinking about the possible reconstruction of networks.

*“…this [intervention] takes it to the next level…… having this chat today got me thinking about, yes, now who among my friends and relatives might be able to be a buddy?” (ID4)*

The mapping and discussion about healthcare support raised awareness of how frequently participants had contact with a healthcare professional and in relative terms how seemingly unimportant they were in terms of self-management support in everyday life. As awareness grew of social network support, the focus on medical-based solutions for long-term condition management was revised.

*“I think you are sort of left on your own, you get an annual check and that’s it. I think there should be more” (ID1)*

Accounts also emphasised the relevance of ‘weaker ties’ [11] such as occasionally seen social acquaintances who did not immediately spring to mind as important network members and were re-evaluated through collaborative discussion. ID5 remembered an acquaintance with skills who provided valued practical assistance and being able to provide reciprocal emotional support was important.

*“There’s one I forgot to put, there’s [friend 1], a master plumber, pipe fitter and god knows what but if I wanted the wash basin taps changed in the bathroom he would be offended if I didn’t ask him and he wouldn’t charge a penny for doing it. He’s the bloke I would turn to… and there’s a reciprocal thing when his marriage went on the rocks which absolutely stunned him it was going that way and he came round and over a coffee he mulled it over”* (ID5)

### Reconstruction of social support

The intervention focussed attention on possible alternatives for support through rehearsing different options to the current situation and seeking new and enjoyable activities. Local opportunities were tailored to preferences and displayed on a map. Often this knowledge was new and unexpected. The network mapping exercise enabled people to readily consider who in their network could help them take up or resume activities or healthy behaviours. This consideration of ‘collective efficacy’ ranged from practical notions—such as ID12 deciding to obtain dietary advice and measuring spoons from her sister who had health expertise—to taking up enjoyable activities with close family members.

*“I suppose what [my wife] and I have talked about is perhaps to try and maybe find somebody to do ballroom dancing or something. It might be fun but I mean the poor girl works her socks off and by the time we’ve finished in the evening she’s knackered.….. if we can find the time then perhaps try and do it, give it a go and see if we like it or not”* (ID15)

What this last quote highlights, is the element of negotiation and time required before new activities can be embarked on. This is considered in the following theme.

### Change and reflections over time

Considerations of how relationships might be negotiated to allow people to take up activities were evident in findings in the 6- and 12-month follow-ups. Some people only required the facilitation of an informational link to identify and access appropriate community resources, but for most, there was a period of inner reflection and a need to renegotiate relationships and develop differing tactics for unforeseen problems (such as timetables at leisure centres not fitting with peoples’ lives and responsibilities). Case study 1 illustrates this.

Case Study 1

**Table:** 

Julie a 66-year-old married woman retired with her husband 5 years ago to the Island where her father and her sister were living. She sees them in a caring capacity on a regular basis. Her grown up children and grandchildren live on the mainland. Since retirement Julie has taken up part-time voluntary work in a charity shop. Julie’s identity is closely linked to helping and supporting other people. Constructive and reflexive engagement occurred over time rather than immediately. Preference elicitation and linking those preferences to relevant network members formed an aspect of the process of reconstructing new alternatives. At T2, Julie demonstrated the navigation of her network and negotiated relationships within it in order to access preferred activities identifying collective activities with acquaintances which she attributed to sustaining her motivation. Some activities were incorporated into maintaining a supportive role towards her father and sister. Her father’s nursing home was opposite a hotel with a swimming pool open to the public all day with more flexible availability than the local leisure centre. This provided Julie with an opportunity to combine visiting her father with her preferred form of regular exercise. Similarly, Julie used to go line-dancing with a friend, but had to give it up when the friend stopped going, as she relied on her friend’s husband for transport. Julie identified a new line-dancing class located near to where her sister lives with weekly visits to support her sister. In addition, Julie negotiated a way to continue attending Slimming World classes when the friend she usually went with stopped attending. When Julie heard that her neighbour’s sister attended a class closer to home, she asked whether she could go with her on the bus. The passage of time allows Julie to review the value of her role in the local Diabetes Support Group, which was onerous and no longer fulfilled her needs. Having an administrative role has kept Julie engaged in the support group for longer than average, as the role of health condition support groups are often only important for a limited length and a specific point in time. Julie decided it was time to step down from her position on the committee which in turn freed up time to increase her weekly voluntary hours in the charity shop.	

### Foregrounding and backgrounding as a means of mobilising the network

The longitudinal approach to this study captured changes in the perceived importance of support from individual network members. Whilst some member positions remained static between T1 and T2, others moved between concentric circles of the network diagram reflecting an increase or a decrease in importance at different time points. Four cases indicated outward movement of healthcare professionals (two GP, one diabetic nurse, one physiotherapist) demonstrating a shift in focus away from healthcare based self-management support as engagement in new activities and community resources increased.

Movement between concentric circles of friends and family was dependent on the fluctuating needs of participants; extension of support as needs increased and contraction as needs decreased. Network mapping over time captured the repositioning of network members as they moved inwards, outwards and sometimes beyond the circle diagram completely. Case study 2 provides an illustration of foregrounding and backgrounding of network members.

Case Study 2

**Table:** 

Susan a 51-year-old woman whose diagnosis with type 2 diabetes 9 months ago, left her feeling shocked, anxious and alone. Susan placed her daughter in the inner circle at T1, as her main source of support. She helped with weight loss and fitness through collaborating with healthy eating and accompanying her to the gym. As Susan lost weight she says she grew in confidence and decided to change to another gym when her favourite class was dropped so was no longer reliant on her daughter for the same level of support. At T2 interview, Susan moved her daughter from the inner to the middle circle (backgrounding). Other examples of network members becoming less important over time included the diabetes nurse and a Facebook group. Although the diabetic nurse played an important role in the initial period following diagnosis, the drop off of frequency of appointments left Susan feeling disappointed and abandoned. Susan decided to move the diabetes nurse from the inner circle (T1) to the middle circle (T2). Likewise, Susan talked about being a member of a Facebook based diabetes support group (T1), but then got annoyed by the group which she described as American-centric and not very ‘uplifting’. This group was backgrounded from the middle to the outer circle. In contrast, Susan placed her partner in the middle circle (T1). She felt that he did not really understand the impact of her recent diagnosis and wanted him to show more interest in her condition and get more involved in her support. At T2, Susan moved her partner from the middle circle to the inner circle to reflect an increase in support (foregrounding). Susan felt that she was able to talk more openly with her partner as her confidence grew, resulting in him becoming more aware, involved and supportive. They are working towards doing exercise together and attending a ‘Sugar Buddies’ social evening as a couple.	

Follow-up allowed the identification of how network members saw themselves becoming more engaged in integrating activities into their everyday lives as well as how making activities fit the responsibilities of existing relationships could open up new horizons and opportunities. ID13 was partially sighted and dependent on his partner driving him to activities, whilst important to him, this dependency was stifling and other network members recognised his need for a personal space. It was a connection to his gym instructor (a weak tie) [11] which enabled this as he recognised that coming to the gym session not only increased the participant’s fitness but also provided an opportunity for him to ‘be himself’ and enjoy a time and space away from a partner he felt dependent on. The gym instructor gave the participant confidence to increase his sessions to twice a week. At T3, his time at the gym was still valued highly—*“it’s brilliant”.* In this interview, he opened up explaining that the gym is ‘me time’ away from his partner. He compared going to the gym to a social club with everyone chatting and joking in a relaxed way. He commented: *“it is ME having the conversation. She (partner) can’t join in”.*

An illustration of fitting change with relationship responsibility is provided by ID07, a carer for a disabled partner. ID07 had started doing table tennis on a weekly basis at T2, a sport she used to do 45 years ago. It worked particularly well because the sports centre was so close to home allowing her to leave her partner on his own for an hour. *“I think that if it was too far away I’d probably think ‘Oh, I can’t be bothered. But because it’s on the doorstep it’s all there.”* Two further activities stemmed from this—a Pilates session advertised at the sports centre and setting up a team with other table tennis players to take part in a local quiz night.

*“It’s useful to see that one thing leads to another. The intervention led to talking to (health trainer), and then talking to (health trainer) led to seeing the poster and that led to the table tennis and now that’s going to lead on to Pilates. So there is a general progression. So yes, I have benefitted quite a bit……..That has happened and you can quite easily track it from one to the other to the other. And here I am talking to you, looking at charts and things……I think overall the whole thing has been a benefit” ID07*

The next theme explores how the role of the facilitator was a key part of the process.

### It has opened doors: importance of a challenging, temporary friend

The intervention made ‘a lot of sense’ to the facilitators because it fitted so well with their role in providing a bridge between healthcare professionals and take-up of social and health-related activities and made their work easier. Feedback from the health trainers included the observation that the intervention *‘felt like part of the service’*. The care navigators went on to use the GENIE circles tool with the majority of their clients as they were particularly interested in identifying people who were isolated and lonely. Comments from their report included:

*The scoring tool originally did not take into account telephone and skype calls which can be very important to people. We have now decided to add telephone calls to the network typology.*

*It does not capture that there are some people who come out on the tool as being diverse as they attend many social groups but when they come home they still feel lonely.*

*It does not recognise professionals who come to be important for them, for example carers who they have had for years they may consider to be friends. Or the postman who will also collect the milk from the local shop.*

The facilitators were recruited from local populations to represent ‘the person next-door’. The perceived lack of status difference presented an opportunity for the facilitator and participant to work together and collaborate at each stage in order to find the most appropriate way forward. Although the facilitator often took the lead, participants played an active role in helping to navigate the intervention’s unfamiliar territory. Personal dynamics and the level of confidence in certain tasks impacted on how the balance of control shifted between facilitator and participant, since the equalising effect also opened up freedom to challenge and be challenged. For example, one participant felt very confident when tackling IT-related issues due to his professional training and wanted to help the facilitator whenever technical difficulties arose.

*“You’ve gone too high…..no just close that……that’s the problem with it going to full screen, with the way you’ve got it set up……when you clicked finish it didn’t either close down the questions or take you to the next page….it didn’t take you to step three…”* (ID04).

Findings indicated that the human presence of a facilitator increased focus, motivation and persistence and elicited a more honest response.

*“I think for me personally, having somebody else there… I thought, I’ve got to think about the answers. Whereas, I think if I was doing it on my own I’d have just put that down and that’s it, that’s done and put it away now and forget about it.…… with* [facilitator] *being there I had to think about the answers and make sure I was….. I feel I was giving her an honest answer”* (ID14)

Facilitators had ongoing working relationships beyond the point of intervention with nine participants who became or were existing clients. These facilitators were often included as network members in the circle diagram at T2 and considered personal friends or ‘buddies’. However, facilitators were expected to have between six and eight visits with each client resulting in potential blurred professional/personal boundaries when associations continued beyond those sessions.

“[Facilitator] *she’s a good friend and it’s nice to have that support….. I never get to see* [her] *much because she’s such a busy lady, isn’t she? I do tell her to come round and she has been round a couple of times but lately she’s gone off the boil. Well, I think with Christmas and New Year and that she’s such a busy lady. It’s trying to fit it all in”* (ID10)

By T3 the ‘removal of the training wheels’ was more apparent. Whilst some like ID10 mourned the loss of a temporary friend, others such as ID04 recognised the need to become less reliant on the facilitator and to begin to mentally move her out of the inner circle thus enacting ways to deal with the loss of network member. Visualising and using the circles became important to him as a way to plan:

*“I’m thinking about other groups, I’m thinking about how to take it forward from where I am. How I can progress into the future. Getting back to work at the moment is in the outer circle but I need to bring it into the middle circle or into the inner circle.…..these ideas are in the outer circle but it’s how they progress towards the inner circle…..I’ve got these networks and I’ve got to sort of start moving them around, making some more important than others and not making myself reliant on somebody. Making myself reliant on myself or looking at going out to parties, this sort of thing, getting myself back into a social environment”*

### Lessons learnt about GENIE delivery

One intention of the study was to record frequency of face-to-face contact with network members. However, the importance of Skype, FaceTime and telephone conversations as valued means of regular contact with close friends and family was not fully anticipated and will need to be considered in the future. An unanticipated finding was the value attached to ‘things’ in the network mapping exercise. Participants placed objects such as walking sticks, mobility scooters, phablets, monoculars, measuring spoons and Wii-fit games as important network ‘members’ in terms of self-management support. Health-related objects such as these become built in sustainable ways into people’s everyday life. In addition, everyday workable objects represent a means of engaging people without involving written information, which has important implications in terms of health literacy.

Lessons learnt about facilitator training suggested a need to place more emphasis on discussing preference options in the final stage of the intervention, since facilitators spent most time on the first stage (network mapping). As the network mapping exercise was so readily incorporated into people’s life and work, it may be worth considering the different elements of the tool as stand-alone interventions.

From the data analysis presented the following process seemingly underpins and characterises how GENIE becomes implementable:

1. *Positive disruption of established self-management practice* through engaging with network map.
2. *Reflection on existing network membership* opens up possibilities for evaluating the present and anticipating, rehearsing and reconstructing self-management differently.
3. *Preference construction and elicitation* are an aspect of the process of reconstructing self-management (establishing preferences of things they would like to do and asking about activities they like that they have stopped doing).
4. *Reversing the focus on the self* to focus on others in a network assists with reflexion and option appraisal that moves beyond individual motivation and existing personal option appraisal.
5. This is enabled through *constructive interpersonal engagement provided from facilitation* where there is perceived lack of status difference (less likely if the facilitators are medics) and the possibility of reciprocity within the discussion (e.g., helping the facilitators to deal with the technology, telling them about groups that are not in the database).
6. *Access to definitive resources* which can be approached via a reliable database of local resources is a precondition for elicitation and moving to connecting to and mobilising broader social capital and self-management options.

## Discussion

This study illuminates the likely mechanisms underpinning the workability of a resource to improve awareness of the nature and potential role of social networks for people with long-term conditions. Key factors relating to health literacy (visualisation in network mapping and findings phase) and ‘fit’ with lay health worker roles differentiate GENIE’s approach from similar interventions. There is a known gap between development of ideas and products and implementation in real-world settings which has been termed the second translational ‘gap’ [29] (the first gap being that between basic research and products). The GENIE approach goes some way to effectively addressing this as the network mapping phase of the intervention is immediately translated into practice by the trained facilitators and utilised with each new client.

Our findings suggest that GENIE works through increasing links to and uptake of social activities. Implemented across a health community, it was found acceptable and appropriate for people living with type 2 diabetes when delivered by trained facilitators in a community setting. Implementation led to an increase in diversity of participants’ networks, greater engagement with community activities and adoption of GENIE by facilitators as part of their routine client assessment.

In contrast to delivery in primary and secondary care settings, health trainers and care navigators embody an ethos that fits and complements GENIE’s composition in terms of collaborative work, user-centred approach and forging links to community resources [13]. Using face-to-face facilitation provided the means for a stronger interpersonal element to develop within the collective work carried out. The equal relationship with lack of status difference was instrumental in allowing this, and facilitation has been shown to be an important aspect of GENIE to support the navigation of options [30].

For people with long-term conditions, the social network approach is a different way of considering the support they have. The building of individual and network capacity is needed to navigate and negotiate relationships and health-related environments to gain self-management support and findings in this study parallel the elements of navigation, negotiation and collective efficacy identified in an earlier review [4]. Findings from this study illustrate the cyclic and temporal nature of network relationship negotiation which requires persistent cognitive and emotional effort to generate collective efficacy. We have shown that engagement with the circle diagrams provides a means of mobilising resources that people can intuitively transpose into real-life relationships in order to help them prioritise and make best use of available support. This new way of thinking—that illness management is more about network relationships and support than personal responsibility—disrupts existing patterns of coping in a non-threatening but powerful way, enhanced by having a visual network image which people can control and alter to help them reflect and take actions to form a more desirable support network. Externalisation which does not focus on the self exclusively enables reflections that are missed or inhibited through introspections. The longitudinal aspect of this study allowed us to capture this change for example, health professionals had less of a central role and weak ties and enjoyed activities came more to the fore.

Being able to see a support network mapped out helps to open up discussions about support and facilitators found this a shortcut to forming a working relationship. It is recognised that people are sometimes overwhelmed with choice which is often only apparent and not actionable, or they do not know where or how to look for support. The multiplicity of databases produced by governmental and health-related organisations is testament to ongoing attempts to provide information about resources and groups—alongside which, there is a growth in new roles for lay people in health-related jobs such as health trainers, care navigators, health coaches, link workers, pathway planners who work to provide a bridge between primary care professionals and social activities [26]. GENIE provides a way of assisting those in this role to help make sense of the choices and availability of support and work with people’s preferences and interests to get them to engage with their local community.

Earlier studies have demonstrated that networks are built and evolve through a process of selection of similar ties (homophily) which are likely to be preferred and sustained over time [31]. Existing contacts are likely to influence and reinforce choices in a process described as contagion operating through replicating practices, modelling on others, upward and downward comparisons [32, 33]. The GENIE intervention builds on these network mechanisms facilitating the process of selection through the identification of new ties. Engagement with community resources can have an indirect effect on self-management and opportunity to reciprocate making further engagement more likely. However, with GENIE, there is recognition that key network ties need to be made visible and reflected upon, so both users and their network members can negotiate what is valuable and acceptable [4].

### Limitations

Results are not generalisable on the basis of health condition or location. The exemplar long-term condition used for the study is type 2 diabetes; therefore, validity of findings in relation to other chronic illness management outcomes is limited. The study is located in an island community comprising a number of distinct inherent features such as geographical marginalisation (families separated by the sea), above national average elderly population living alone and at risk of isolation and a predominantly white British population [34]. Video-elicitation did not work well as a method due to technical complexities, the method was adapted successfully through use of researcher observation notes to elicit ‘thinking aloud’ associated insight.

## Conclusions

GENIE is an evidence-based health intervention which can help to change people’s established way of thinking about self-management support and offers a route to change practice and behaviour patterns in everyday life. The intervention achieves this by raising awareness of social networks, using social networks to engage people in reflection of their support and access to further resources and by improving patient engagement through strengthening existing individual and community networks. The intervention ‘fit’ with real-world environments and the working remit of community-based lay health workers points to potential for adoption and integration into healthcare policy and practice.

## Additional files

Additional file 1: **Premises for a social network approach to self-management focussed on engagement and preference. **(DOCX 38.2 kb)

Additional file 2: **Training outline. **(DOCX 16 kb)

Additional file 3: **Use of normalisation process theory as framework for analysis of video in case study 2. **(DOCX 23.3 kb)
