# Supplementary material for: Implementing a social network intervention designed to enhance and diversify support for people with long-term conditions. A qualitative study
Source: Implement Sci. 2016 Feb 29;11:27. doi: 10.1186/s13012-016-0384-8 (PMC4772323; doi:10.1186/s13012-016-0384-8)
Supplement: Supplementary file 1 — Premises for a social network approach to self-management focussed on engagement and preference. (DOCX 38.2 kb) [file 13012_2016_384_MOESM1_ESM.docx]

**Additional file 1**

**Premises for a social network approach to self-management focussed on engagement and preference**

***Prioritising social support over specific illness support***

Prioritising social engagement over engagement with narrowly defined illness specific support is likely to be more effective in supporting self-management. Highly directed approaches to self-management such as the Expert Patients Programme tend only to reach a small minority of people and not the deprived populations most likely to need such support.[1] Prioritising improvements in self-efficacy could also preclude positive engagement with one’s material and social needs.[2] More broadly, non-coercive and non-intrusive methods of engagement have been found to be more effective in engaging users in fields as diverse as the provision of parental support for children with type 1 diabetes, people with longer term mental health problems and young offenders.[3-5]

***Directive approaches fail to engage***

Highly directed approaches are likely to fail because of barriers that are often hidden or remain latent and not thought about by users or facilitators. Barriers to taking up new practices often involve relationships, for example not having anyone available to do something new with can be intimidating or existing relationships may have to be renegotiated to allow time and space for new activities. New social engagement can create difficulties within networks, for example, someone who is cared for or is a carer for others may have a network which lacks the capacity to step in to provide regular support. Resources are often a problem, such as cost or lack of transport.[6, 7]

***Engagement through opportunities for reciprocity***

Another aspect of prioritising user preference is construing the user as a capable and willing to reciprocate participant rather than as someone who is exclusively in need of support. Opening possibilities for user involvement in a meaningful and non-prescriptive manner, and to the extent to which they are capable and willing to contribute, has also been shown to increase the likelihood of user engagement with self-management interventions. This could be as a part of the intervention process or more broadly understood volunteering and altruism.[8]

***General appeal of network mapping technique***

The network mapping technique has been applied widely in disciplines such as counselling, family therapy, education, social work, in communicating with children capitalising on their interest in drawing, allowing non-verbal communication and reducing power differentials, with adults with learning disabilities, and in person-centred planning.[9-13] The network mapping technique has been found to create a process that is open and where insights that might be taken for granted or overlooked, can be reflected on, and acted upon.[11]

***Reducing complexity and information overload***

While the potential of the voluntary sector to provide resources, emotional, social and practical support for people with long-term conditions is widely acknowledged it is poorly understood and specified.[14, 15] In engaging with available resources users have to deal with a set of complexities arising from information, navigation and negotiation overload, which is likely to be most difficult to achieve for deprived populations and those with low health literacy. Cognitive and sensory overload has been shown to be counterproductive for learning, social engagement and social support and thus may constitute a considerable obstacle to engagement.[16, 17]

1. Kennedy A, Reeves D, Bower P, Lee V, Middleton E, Richardson G, Gardner C, Gately C, Rogers A: **The effectiveness and cost effectiveness of a national lay led self care support programme for patients with long-term conditions: a pragmatic randomised controlled trial**. *Journal of Epidemiology and Community Health* 2007, **61**:254-261.

2. Kennedy A, Rogers A, Crossley M: **Participation, roles and the dynamics of change in a group-delivered self-management course for people living with HIV**. *Qualitative Health Research* 2007, **17**(6):744-758.

3. Young MT, Lord JH, Patel NJ, Gruhn MA, Jaser SS: **Good Cop, Bad Cop: Quality of Parental Involvement in Type 1 Diabetes Management in Youth**. *Current Diabetes Reports* 2014, **14**(11).

4. Taylor TL, Killaspy H, Wright C, Turton P, White S, Kallert TW, Schuster M, Cervilla JA, Brangier P, Raboch J *et al*: **A systematic review of the international published literature relating to quality of institutional care for people with longer term mental health problems**. *BMC Psychiatry* 2009, **9**(55).

5. McGuire J: **Comparing coercive and non-coercive interventions**. In*.* London: Centre for Crime and Justice Studies King's College; 2010.

6. Vassilev I, Rogers A, Kennedy A, Koetsenruijter J: **The influence of social networks on self-management support: a metasynthesis**. *Bmc Public Health* 2014, **14**(1):719.

7. Park NS, Zimmerman S, Kinslow K, Shin HJ, Roff LL: **Social Engagement in Assisted Living and Implications for Practice**. *Journal of Applied Gerontology* 2012, **31**(2):215-238.

8. Kahana E, Bhatta T, Lovegreen LD, Kahana B, Midlarsky E: **Altruism, Helping, and Volunteering: Pathways to Well-Being in Late Life**. *Journal of Aging and Health* 2013, **25**(1):159-187.

9. Attneave C: **Core network intervention: An emerging paradigm. Journal of Strategic and Systemic**. *Journal of Strategic and Systemic Therapies* 1990, **9**:3-10.

10. Whyte B, Fraser D, Aitken V, Price G: **Interactive group activity: a socially mediated tool for opening an interpretive space in classroom research**. *International Journal of Qualitative Studies in Education* 2012, **26**(8):1019-1040.

11. Hill M: **Network Assessments and Diagrams: A Flexible Friend for Social Work Practice and Education**. *Journal of Social Work* 2002, **2**(2):233-254.

12. Hill M, Laybourn A, Borland M: **Engaging with Primary-aged Children about their Emotions and Well-being: Methodological Considerations**. *Children & Society* 1996, **10**(2):129-144.

13. Seed P: **Introducing Network Analysis in Social Work**. London; 1990.

14. Milligan C, Gatrell A, Bingley A: **'Cultivating health': therapeutic landscapes and older people in northern England**. *Social Science & Medicine* 2004, **58**(9):1781-1793.

15. Ziersch AM, Baum FE: **Involvement in civil society groups: Is it good for your health?** *Journal of Epidemiology and Community Health* 2004, **58**(6):493-500.

16. Eppler MJ, Mengis J: **The concept of information overload: A review of literature from organization science, accounting, marketing, MIS, and related disciplines**. *Information Society* 2004, **20**(5):325-344.

17. Misra S, Stokols D: **Psychological and Health Outcomes of Perceived Information Overload**. *Environment and Behavior* 2012, **44**(6):737-759.
