# Supplementary material for: Implementing a social network intervention designed to enhance and diversify support for people with long-term conditions. A qualitative study
Source: Implement Sci. 2016 Feb 29;11:27. doi: 10.1186/s13012-016-0384-8 (PMC4772323; doi:10.1186/s13012-016-0384-8)
Supplement: Supplementary file 2 — Training outline. (DOCX 16 kb) [file 13012_2016_384_MOESM2_ESM.docx]

**Additional file 2**

**Training Outline**

| **Background**   - Traditional self-management support delivered through primary care - Role of social networks - Evidence and background for interventions to enhance network support - Big picture aims |
| --- |
| **Discussion** |
| **Demonstration of the online version of GENIE**  How to use GENIE (online and paper-based)  Process of tailoring: how does GENIE work:   - Needs/preferences - Mapping networks and network type - Establishing links to existing resources - Facilitating use of resources and links to network support - How to develop rapport - Looking for clues and working with personal preferences and need |
| **Coffee break** |
| **Activity – using the GENIE website**   - In pairs, lead your partner through the process of creating a personal support network. Use a real example, either yourself or someone you know well who has a long-term condition. - For each person or organisation on the network, add their relationship and the frequency of contact - Discuss the GENIE questionnaire and select the appropriate options - Discuss the available options for linking to local resources and potential support from network members |
| **Lunch** |
| **Discussion: What was it like to use GENIE? Problems, comments** |
| **Introduction of the GENIE workbook** |
| **Activity – Building a database**   - In pairs, discuss three organisations in your areas - Use your existing knowledge and internet in order to fill in the data entry form provided |
| **Feedback and introduction of the online data entry form** |
| **Tea break** |
| **Activity – Online data entry**   - In pairs, use the organisations identified in the paper based exercise and input the data into the online form |
| **Discussion and demonstration using the newly added online resources** |
| **Group discussion– who will deliver GENIE** |
