# Supplementary material for: Implementing a social network intervention designed to enhance and diversify support for people with long-term conditions. A qualitative study
Source: Implement Sci. 2016 Feb 29;11:27. doi: 10.1186/s13012-016-0384-8 (PMC4772323; doi:10.1186/s13012-016-0384-8)
Supplement: Supplementary file 3 — Use of normalisation process theory as framework for analysis of video in case study 2. (DOCX 23.3 kb) [file 13012_2016_384_MOESM3_ESM.docx]

| **Additional file 3: Use of Normalisation Process Theory as framework for analysis of video in case study 2** | | | | |
| --- | --- | --- | --- | --- |
| **COHERENCE**  **Meaning of practice: sense-making work**  Does the practice make sense? What is the work involved? | | | |  |
| **Components** | **Questions to consider** | | **Questions used in GENIE evaluation** | **GENIE** |
| Differentiation | Does the participant (P) understand what s/he is being asked to do?  Does the P recognise this approach as being different from existing ways of working?  Does the P understand the purpose of self-management support (SMS)?  Does the P attribute a specific meaning to SMS?  Does the P understand the purpose of each step in the intervention?  Does the P understand the role of the facilitator? | | Achieved understanding of what information the facilitator required  Indications that this is a different or new way of thinking about what help and support they have or need | Achieved understanding quickly – nodding – but had to refer to written list when asked to populate circles  A new way of thinking – not used to thinking about herself in relation to the support she gets from others as used to doing things on her own therefore has not considered involving others in decisions and activities  Aware of self-management – shared purpose in deciding who she does or could seek help/support from  Growing awareness in the types of people/groups she could relate to eg the work-based weight monitoring group was only mentioned at the end  Diagram not seen as relevant to start with – an indicator of what help she needed to an outsider – if she hadn’t put anyone in the middle, then she would have needed help |
| Communal specification | Does the P have a shared sense of purpose (with the facilitator)?  Does the P have a shared understanding of working together (with the facilitator)?  Does the P understand the steps s/he needs to take to assist in using this approach? | |  |  |
| Individual specification | Does the P understand what this approach requires of him/her as an individual?  Does the P understand their specific tasks and responsibilities?  Does the P identify their personal role in using GENIE? | |  |  |
| Internalisation | Does the P understand the value of each step of the intervention?  Does the P understand the overall value of GENIE?  Does the P identify any personal benefit in adopting the GENIE approach?  Does the P see the approach as helpful and/or relevant to them as an individual? | | Why do you think the diagram of names might be helpful? |  |
| **COGNITIVE PARTICIPATION**  Commitment & engagement: relational work  Who does the work? | | | |  |
| Initiation | Has the P shown willingness to support the process to implement GENIE?  Has the P engaged with the facilitator to drive the implementation forward?  Did the P think the intervention was a good idea?  Was the P prepared to invest time/energy/work in GENIE? | Got ‘buy in’ (i.e. able to clearly identify people and activities relevant to SMS | | Buy in as able to think about the gaps in her support – highlighted her vulnerability and the need to meet others with diabetes.  Legitimate because of withdrawal of support from NHS following diagnosis – the 6 weekly appointments dropped to annual  Isolated – daughter main support but not living with her – doesn’t talk to son who does live with her – and partner hard to engage as doesn’t understand  Hard to know where to place partner – does not want to demean importance, but not supportive (in middle circle)  Facebook resonated as source of support, but those responding were American so lack of relevance  The results were valued and the activities were relevant – local diabetes group learnt about and intentions to join |
| Enrolment | Has the P engaged with the GENIE process?  Does the P work together with the facilitator in discussing the GENIE questions?  Has the P made any adaptations to their personal routine?  Has the P assisted in the reorganisation process leading to implementation? | Personally relevant (i.e. they were able to identify that they needed support from others to help with day to day management OR the opposite – they needed no support from others)  Indicated awareness about type of network – that they were isolated or well-supported  Indicated awareness of gaps in SMS – any mention of the types of support work they needed help with  Indicated problems with managing their condition  Indicated barriers or problems with network members or situations  Indicated awareness there had been a change in activities (e.g. activities they used to do)  What did you understand your type of network to mean?  How could network members (or anyone else) help you take up activities (again)? | |  |
| Legitimation | Does the P believe it is appropriate for them to be involved in implementing GENIE?  Does the P believe s/he can make a valid contribution?  Did they see the point easily? | There are questions of relevance to them  Indications that the results are of value to them  Did you learn anything new about yourself?  Did you learn about any new activities or organisations locally? | |  |
| Activation | Has the P identified actions in order to sustain the use of GENIE?  If so, has s/he done this individually or with others?  Has the P taken steps to stay involved in the use of GENIE? | Indicated they may want to participate again in that activity  Able to prioritise relevance of activities  Indicated others in their network they could do activity with/work with  Able to state how they might put preferences into action  What do you think you will do now? | |  |
| **COLLECTIVE ACTION**  Interaction with already existing practices: operational work  How does the work get done? | | | |  |
| Interactional workability | Does the new practice change relationships in a way that is workable and acceptable to parties involved | How was the facilitator helpful? How was the facilitation not helpful? How could it be improved?  What difference do you think it would have made if you had done this on your own? | | Thought facilitator not needed, could do it on her own online  Will go to health and wellbeing weekend organised by local diabetes group – facilitator helped with this motivation  Worked with facilitator to decide how to position people and groups |
| Relational integration | Build and maintain confidence in the relevance and value of the new practice | Indications that the facilitator has helped them find new activities of value and motivated them to make connections | |  |
| Skill set workability | Are Ps able to acquire the new set of skills and are they able to re-distribute and change the existing ones |  | |  |
| Contextual integration | How does this new practice fit into the broader context, e.g. the need to redistribute time and resource allocation |  | |  |
| **REFLEXIVE MONITORING**  Understanding & assessing: appraisal work  How is the work understood and assessed by actors implicated in it? | | | |  |
| Systematization | Seeking to determine how effective and useful is the new practice | Who else do you think this type of assessment would help? | | Able to identify where GENIE could be operationalised – at special diabetes sessions organised for newly diagnosed patients by NHS  Reflected on other places/groups of people she could talk about her diabetes with eg weight group at work |
| Communal appraisal | Working together with other Ps to evaluate the worth of the new practice |  | |  |
| Individual appraisal | Assessing how the new practice impacts on other existing tasks |  | |  |
| Reconfiguration | Have they (as individuals and in relation to other network members) changed or tried to change their practices. |  | |  |

**Comments**

This process was challenging:

- Lack of people to do things with
- Hard to place partner in the diagram
- Highlights lack of NHS support
- Vulnerability as no collective efficacy
- New skills to engage support from others hard to develop as so used to being independent

Although she thought the facilitator could be bypassed – this was a vital role in connecting her to support and highlighting sensitively her lack of support. Her home was where she felt most vulnerable and unsupported
